# Prevention and Cure

**Published:** 1922-01

**Authors:** S. H. Daukes


### PREVENTION AND CURE.

By S. H. DAUKES, O.B.E., M.B., D.P.H., D.T.M. and H.

IT IS commonly reported that in certain parts of the world it is customary to pay the family doctor when there is no sickness, but to stop payment when treatment is necessary. Such a custom must give rise to unending legal difficulties, more especially in an age when functional disease is so prevalent; it does, however, call attention to an ethical aspect of medical work which there is a tendency to overlook.

The family physician is called in to see a child who has a sore throat; he examines the patient and comes to the conclusion that he is possibly dealing with a case of diphtheria. A swab is taken and appropriate treatment is instituted. The doctor's work, however, is not ended; he must go further than this and advise isolation of the patient until the diagnosis is settled, in order to safeguard the other children in the family or school. Moreover, should the swab prove to be positive, the Schick test will enable him to recognise susceptible individuals who can be further protected by the injection of anti-toxin. Automatically his work has divided itself into two parts—treatment and prevention; the first he has been asked to undertake, the second is a moral obligation which he has taken upon himself. Four cases of diphtheria would be far more profitable to him than one, but such a consideration has no weight in the realm of medical ethics.

Passing to the wider field of general preventive medicine, we realise that vaccination and general hygiene have practically removed from the doctor's visiting list two diseases—smallpox and typhoid fever—which at one time were a source of much financial gain. It is, in fact, the duty of the medical profession to exert every effort to destroy its own means of livelihood by preventing disease. Prevention is better than cure, but what the eye does not see the heart does not grieve over, and it would be a very enlightened public which could appreciate the man who warded off a possible evil equally with the man who removed an actual disability.

Year by year the list of preventable diseases grows and the organised efforts to prevent disease increase. We live in an age of specialities, an age of 'ologies and 'ographies, when the general practitioner is increasingly dependent upon specialists and institutions. At such a time the danger of the water-tight compartment becomes imminent and there is a tendency to narrow-mindedness.

The intensive specialisation of public health work has tended to dissociate the clinician from the sanitarian, so that at times the practitioner has resented what has been regarded as an intrusion into his private domain. Moreover, there is a liability amongst the public to regard the science of prevention as synonymous with the management of drains and dust-bins. Any such misunderstanding must be detrimental to progress.

All prevention must commence at the bedside of the sick person, the domain of the clinician, with whom rests the responsibility for diagnosis, notification and treatment, but from that centre it has many ramifications into the realms of laboratory investigation, designing, planning, engineering, construction and legislation, whither the clinician would have little leisure or inclination to follow it. There must be intimate and cordial co-operation between clinical and preventive medicine.

In " tropical medicine " the need for such a liaison is specially well seen. Here the clinician and hygienist meet on common ground in the laboratory and at the bedside, and in co-operation each finds a solution for his problems, be they diagnostic or prophylactic. Moreover, in the tropics the clinician must in self-protection be a hygienist, whilst the hygienist must be a clinician, seeing that much prevention depends upon efficient treatment.

The value of wholesale and intensive treatment as a prophylactic measure is being urged in many of the diseases of hot climates. The French have attempted to cope with the menace of sleeping sickness by the atoxylisation of large numbers of infected and suspected natives. The Rockefeller Foundation is attempting to stamp out ankylostomiasis by wholesale treatment and education. The treatment of malaria carriers, not only as a curative, but also as a prophylactic measure, has been repeatedly advocated; whilst the effective introduction of antimony preparations in the treatment of bilharziasis offers some hope of dealing with this widespread disease at the fountain head. Obviously where such methods are adopted or suggested there can be no dividing line between the clinician and the hygienist.

At the present time those who work in temperate climates can learn much from a consideration of the diseases which we are apt to label " tropical." In spite of Kipling's well-known lines,' we must acknowledge that daily the East and West are approximating so far as disease is concerned. The war has reminded us that such diseases as malaria and dysentery are not essentially tropical and that, under favourable circumstances, indigenous cases may occur in England. Rapid modes of transport, more especially aerial, present many possibilities with regard to disease distribution in the future. Our ports are constantly in contact with the problem of tropical disease, and a Port Health Officer must be an able clinician as well as a skilled sanitarian. At the best, the term " tropical disease " is more utilitarian than scientific, and most textbooks dealing with this subject include in their list of diseases many which are by no means confined to the tropics, but which find an unsympathetic reception and uncongenial home in lands where a sanitary conscience and an efficient Public Health Service exist.

Plague, on many occasions, has been epidemic in the British Isles; it is now only a sporadic visitor. At one time malaria was endemic in certain districts; it is now rare except when carriers are imported into the country in large numbers, as they were during the late war. Typhus has been responsible for many

**Prevention and Cure—(*cont.*).**

epidemics in our Islands ; indeed, this disease, which is generally included in tropical disease textbooks, is quite as much a menace to cold climates as to hot. Dysentery, both amœbic and bacillary, occurs in England, and recent work has shown that amongst the healthy population many individuals are carriers of the *Endamœba histolytica.* Pellagra has always been considered to belong to the realm of tropical medicine, but it has been proved that many cases have occurred in the British Isles, and have languished unrecognised in our asylums, whilst during the last twenty years numerous cases of this disease have been diagnosed in the more temperate parts of America. Leprosy is always included amongst the tropical diseases, but at one time it was common in England, and it still persists in Norway ; in fact, its distribution is practically universal.

A few diseases, owing to the conditions necessary for their special mode of distribution, are more definitely confined to hot countries. Sleeping sickness, which is conveyed by the tse-tse fly, is confined to the parts of Africa where the incriminated species of fly exist. Yellow fever, except for very sporadic raids on Western ports, is confined to areas which may be classified as tropical, but in this disease the recent work of Noguchi on the *Leptospira icteroides* makes us think of the close relationship of this organism to the *Leptospira icterohæmorrhagiæ*—the cause of infectious jaundice—which is so prevalent in our country rats, and occurs also in our London sewer rats. The form of malaria due to *Plasmodium falciparum* is rare, except in hot climates, owing to the developmental requirements of the mosquito stage of this parasite, but recently an indigenous case was reported in England.

We see, then, that the distribution of disease is very largely determined by the hygienic state of a community, and a sound knowledge of tropical disease is incumbent upon all medical men who would study medicine from every point of view. The lessons learnt abroad may well be utilised at home, and one of the greatest of these lessons is that which teaches the value of co-operation between the clinician and the hygienist.

A Museum of Tropical Medicine and Hygiene* has been established at the Wellcome Bureau of Scientific Research, which is arranged so as to emphasise the essential unity between these two great branches of medicine. An introductory section is devoted to the question of hygiene in the tropics ; here by models, photographs and paintings are shown the essentials of prophylaxis in hot countries—water supply and purification, conservancy, housing, clothing, food supply, disinfection, &c. Other sections are devoted to the various diseases which are regarded as mainly of tropical interest. These diseases are grouped according to their mode of causation or transmission, a system of classification which is of special value to the hygienist in that each group has its own special line of prevention ; for example, in one section are grouped together the mosquito-borne diseases—malaria, yellow fever, dengue and filariasis. Here prevention depends mainly upon anti-mosquito measures. Another section is devoted to excremental infections—cholera, dysentery, ankylostomiasis, bilharziasis, &c. Here prevention is chiefly a question of water supply and good conservancy. Another section includes the various food deficiency diseases—beri-beri, scurvy and pellagra, and so on. Each disease is dealt with fully and in a graphic manner. To take as an example the most important of tropical diseases—malaria. After a historical and geographical survey, there follows a detailed consideration of the etiology of the disease, illustrated by photographs and paintings, dealing with the parasite, mosquito vectors and their breeding places. The pathological and clinical aspects are then fully dealt with and illustrated by photographs, paintings and specimens. The last part of the section is given up to prevention and treatment. Although prevention is shown in this place, it must be remembered that it is intimately concerned with the question of etiology. The consideration of prevention includes propaganda work, investigation, destruction of mosquitoes, both adults and larvæ, drainage, filling in, municipal undertakings, and finally domestic and personal measures, including the use of the mosquito net and prophylactic quinine.

Each disease in turn is dealt with in a similar manner, every point of importance being fully illustrated. By such a system the clinical and prophylatic aspects are balanced, and it is possible to survey the disease in its entirety.

Without hesitation one can state that in the tropics the sanitary expert must be a good clinician and *vice versa*, and it is probable that this is also true in our own country, where experience in general practice must be a valuable asset to anyone who hopes to undertake Public Health work in a sympathetic and co-operative spirit.

* 25/27, Endsleigh Gardens, Euston Road, N.W.1.

---

### CIVIC STUDY VISIT TO PARIS.

The Civic Education League is organising a visit to Paris for the purpose of affording those interested in sociology, regional survey, industrial conditions, local government and the civic arts an opportunity of first-hand study under conditions of a character different from our own. A detailed study of Paris in so short a time will not be possible, but the programme for the period of the visit will be very varied. Mr. Alexander Farquarson, M.A., is in charge of the educational side of the tour, and will take the region as a basis of study. Visits to parliamentary, local government, educational, and other institutions will be made. Industrial centres, art galleries and churches will be visited. Lectures will play an important part in the programme.

The League has been fortunate in securing the use of a small residential college situated in the Latin Quarter. Students will be resident there during the full time of their stay. The general arrangements of the tour are in the hands of Miss Margaret Tatton, Civic Education League, Loplay House, 65, Belgrave Road, S.W. 1, from whom all particulars may be obtained.

---

Sir Garrod Thomas, of Newport, who is Vice-President of the University College of Wales at Aberystwyth, has intimated his intention of handing over to the college at an early date freehold property in the neighbourhood of Llanarth, Cardiganshire, consisting of farms valued at £8,000, for the purpose of creating a fund for the encouragement of post-graduate work in the departments of chemistry and physics at the college.

